# DARTPHROG: A Superscalar Homomorphic Accelerator

**DOI:** 10.3390/s25165176

**Published:** 2025-08-20

**Authors:** Alexander Magyari, Yuhua Chen

**Affiliations:** Department of Electrical and Computer Engineering, University of Houston, Houston, TX 77204, USA; yuhuachen@uh.edu

**Keywords:** post quantum cryptography, PQC, fully homomorphic encryption, CKKS, BGV, modular reduction, security

## Abstract

Fully Homomorphic Encryption (FHE) allows a client to share their data with an external server without ever exposing their data. FHE serves as a potential solution for data breaches and the marketing of users’ private data. Unfortunately, FHE is much slower than conventional asymmetric cryptography, where data are encrypted only between endpoints. Within this work, we propose the Dynamic AcceleRaTor for Parallel Homomorphic pROGrams, DARTPHROG, as a potential tool for accelerating FHE. DARTPHROG is a superscalar architecture, allowing multiple homomorphic operations to be executed in parallel. Furthermore, DARTPHROG is the first to utilize the new Hardware Optimized Modular-Reduction (HOM-R) system, showcasing the uniquely efficient method compared to Barrett and Montgomery reduction. Coming in at 40.5 W, DARTPHROG is one of the smaller architectures for FHE acceleration. Our architecture offers speedups of up to 1860 times for primitive FHE operations such as ciphertext/plaintext and ciphertext/ciphertext addition, subtraction, and multiplication when operations are performed in parallel using the superscalar feature in DARTPHROG. The DARTPHROG system implements an assembler, a unique instruction set based on THUMB, and a homomorphic processor implemented on a Field Programmable Gate Array (FPGA). DARTPHROG is also the first superscalar evaluation of homomorphic operations when the Number Theoretic Transform (NTT) is excluded from the design. Our processor can therefore be used as a base case for evaluation when weighing the resource and execution impact of NTT implementations.

## 1. Introduction

Recently, data breaches have plagued the cybersecurity community. While encryption standards continue to advance, so have hacking and social engineering techniques. In addition, e-commerce and social platforms have been exposed for selling client data to third parties. These two points highlight the need for zero-trust computing, where unencrypted client data is never shared with a server. In this manner, personal data cannot be stolen or sold, as the only one with access to said data will be the client themselves. This move will require a shift from conventional asymmetric cryptography, as new tools, such as machine learning and other cloud servers, continue to usher in the need for clients to send their data to remote systems.

**Figure 1:**
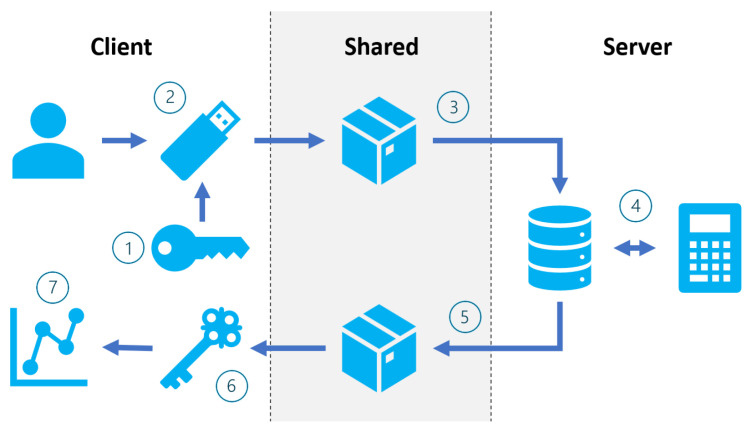
A pictorial representation of the data sharing in homomorphic encryption.

A potential solution for this problem is homomorphic encryption (HE). HE allows for performing arithmetic on encoded and encrypted plaintexts by operating on their respective ciphertexts and has seen applications ranging from simple arithmetic to machine learning applications [1,2]. This is a significant deviation from the more common asymmetric cryptography, in which a private and public key combination is used to secure data *only* between a client and server, where the server acts on the decrypted data from the client. In the case of HE, the client can share data with the server without ever exposing their unencrypted data, which is incredibly valuable in a society where public data breaches are a looming threat. This paradigm, which we illustrate in Figure 1, is atypical of conventional cryptography.

The steps taken throughout Figure 1 are as follows:

1. The client generates a public and private key pair.
2. The client encrypts their data with a public key.
3. The public key and encrypted data are sent to a server to be homomorphically evaluated.
4. The server evaluates the encrypted data without ever revealing the plaintext.
5. The evaluated data is sent back to the user.
6. Using the previously generated private key from Step 1, the user decrypts the data.
7. The user is able to access the calculated plaintext.

Despite their benefit, homomorphisms come with a few significant drawbacks that must be considered [3]. The first is that, in order to ensure security within a ciphertext, noise is added to the encrypted plaintext. It is essential that this noise can be removed from the unencrypted ciphertext, as too high of a noise level will make the data unrecoverable, even for those with the secret key. As the ciphertext undergoes various arithmetic operations, noise accumulates in either a linear or exponential factor, depending on the operation [4].

There are a few different methods to approach the noise level within HE. These methods are used to categorize homomorphic encryption algorithms:

1. Somewhat Homomorphic Encryption (SHE) limits the number of arithmetic operations depending on the chosen scheme. This type of HE is only suitable for small, fixed-depth computations. An example of this is the Boneh–Goh–Nissim HE algorithm, which allows for a large number of additions and a single multiplication [5]. Another classic example is the Paillier cryptosystem which allows for unlimited additions and no multiplications [6].
2. Leveled Homomorphic Encryption (LHE) schemes guarantee circuits of a certain depth *L* before the noise obscures the data from the client. In this way, the level *L* is a parameter chosen by the user, rather than one dictated by the scheme, as in SHE. These algorithms may be beneficial to users who have a known circuit to evaluate, allowing them to avoid the overhead of Fully Homomorphic Encryption. One of the first examples of Leveled Homomorphic Encryption is the Brakerski, Gentry and Vaikuntanathan (BGV) algorithm, which accelerates HE by avoiding the overhead of bootstrapping [7]. Further, LHE avoids the circular security assumption, which refers to the belief that encrypting a secret key under itself does not compromise the overall security of the system.
3. Fully Homomorphic Encryption (FHE) allows for the evaluation of a circuit of arbitrary depth. This is made possible by “refreshing” the ciphertext through bootstrapping, a process that removes the noise from the cipher without fully decrypting it [8]. Through bootstrapping, the server can operate on a cipher until it becomes almost too noisy to recover the data, refresh the cipher, and keep operating. There are many popular FHE schemes, including BGV with bootstrapping, Brakerski/Fan-Vercauteren (BFV) [9], Cheon-Kim-Kim-Song (CKKS) [10], and Torus Fully Homomorphic Encryption (TFHE) [11].

The second drawback of HE is that they are computationally expensive. Plaintext data is encoded into large polynomials, ranging from degree $2^{12}$ to degree $2^{18}$. In addition, the difficulty of breaking an HE scheme is intentionally exacerbated by utilizing large coefficients, ranging up to $2^{10}$ bits per coefficient. This can result in each ciphertext easily reaching tens of megabytes. We discuss two methods for approaching these large ciphertexts later in Section 2.

### 1.1. Homomorphic Encryption in Sensor Networks

While Homomorphic encryption can be applied to all environments of data sharing, it can be a powerful tool specifically for wireless sensor networks. One of these reasons is due to the data sharing nature of sensor networks, as sensors data can be fused between sensors, and the data is vulnerable during a transmission. For example, a work by Kumar et al. explores the end-to-end homomorphic encryption for wireless sensor networks (WSNs) [12]. Kumar’s work allows for homomorphic aggregation functions such as count, sum, and average. By evaluating the data homomorphically, the sensor information is protected during transmission, against wormhole attacks. This method of utilizing homomorphic encryption for secure data transactions is also presented in a work by Li et al., which uses a similar method for data transmission [13]. Li’s method, relying on Paillier Homomorphisms, also encrypts data between the source edge nodes and the receiving sink.

Further, a work by Ifzarne et al. explores making homomorphic encryption more efficient for WSNs by compressing the ciphertexts before transmission [14]. This manner of data handling allows for secure data transmission, while the reduction in payload size allows for power-saving in data transmission. In their model, data compression is a low-power computation, while data recovery, a computation that requires significantly more power, is computed on the sink side, where power is abundant.

Another work, by Sheela et al., [15] takes this concept for WSNs one step further. Sheela’s work also relies on homomorphisms for data security, but operates on the data in a different manner. The encrypted data is used to train an artificial intelligence model, all without revealing the sensor data. This could be particularly useful in environments where massive amounts of data are required to train a model, but privacy is an important factor. Examples of this could be in the medical field, or using images from private devices, such as cell phones.

Finding the balance between an efficient and power-saving FHE implementation is a difficult task, especially in the sense of WSNs, where power saving is a high priority. With this in mind, we sought to explore the necessity for an important, but non-essential, function in FHE. Removing unnecessary functionality could save on static power consumption while negatively impacting dynamic power consumption. We explore those contributions and our exploration method in the next section.

### 1.2. Contributions

In this work, we describe our FHE accelerator, the Dynamic AcceleRaTor for Parallel Homomorphic pROGrams (DARTPHROG, DP). DP is the first accelerator of its kind that allows for the superscalar instructions. By implementing multiple Arithmetic Logic Units (ALU) primitives, instructions for different polynomial registers can be computed in parallel without a reduction in throughput. Limited by the card size, DP does not integrate the common bootstrapping operation and instead, defers that responsibility to the host processor.

Furthermore, the DP paradigm proposes storing FHE ciphertexts and plaintexts as individual registers, allowing for simplified microcode. DP is also the first FHE accelerator to implement the Hardware Optimized Modular-Reduction (HOM-R) system [16], showcasing the low-latency, low-area requirement for this new Modular-Reduction technique that is essential in FHE.

The DP platform integrates a microcode similar to THUMB, but with a more limited instruction set. This system deemed DARTPHROG ISA, DISA, includes eight ciphertext registers, which are stored directly into High Bandwidth Memory 2 (HBM2) memory to allow for massive bus parallelism, large coefficients, and an expansive coefficient set. In addition, our acceleration platform offers the following instructions:

1. Direct Memory Access (DMA) Memory Load
2. DMA Memory Store
3. Ciphertext/Ciphertext Addition
4. Ciphertext/Plaintext Addition
5. Ciphertext/Ciphertext Subtraction
6. Ciphertext/Plaintext Subtraction
7. Ciphertext/Ciphertext Multiplication
8. Ciphertext/Plaintext Multiplication

FHE is often accelerated by the Number Theoretical Transform (NTT), which is a method described later in Section 2.1. A significant increase in throughput for polynomial multiplication is demonstrated by the NTT. Unfortunately, a significant amount of resources is required by the NTT—so much so that some NTT designs require their own Field Programmable Gate Array (FPGA).

The design decision behind balancing NTT resources with the resources required for the rest of the FHE algorithm has yet to be explored. The typical approach is for the majority of the available resources to be allocated for the NTT implementation, which restricts the capabilities for other FHE operations such as ciphertext/polynomial multiplication and addition. A foundation for the decision-making process is offered through the evaluation of the speedup of a digital design that does not implement the NTT. Better reasoning for reducing the non-NTT area can be achieved, as the trade-off between the NTT and non-NTT speedup can be thoroughly evaluated.

The rest of the paper is organized as follows. Section 2 gives a high-level overview of the mathematics required to understand the fundamentals of HE. This section also covers recent advances in HE acceleration. Section 3 covers the implementation of the DARTPHROG accelerator, as well as the DISA implementation and programming methodologies. Section 4 presents the results of DARTPHROG. Section 5 compares our accelerator with other accelerators and HE software, such as Microsoft SEAL. Finally, Section 6 concludes the paper.

## 2. Background

There are multiple approaches to accelerating Homomorphic Encryption. However, before we dive into those implementations, we discuss the necessary mathematics required to describe the functionality of an accelerator.

### 2.1. Preliminaries

The multiple variants of FHE, which we describe later in the section, are all derived from similar mathematical properties. Here, we give a basic, high-level overview of some of the topics and some mathematical definitions. This subsection will serve to better explain our architectural choices for DARTPHROG. We begin with some necessary number theory definitions:

1. Group: A group *G* is a set with a single associative operation, denoted · with the following properties:
  (a) For all a,b∈G, the result of a·b is also in *G*.
  (b) For all a,b,c∈G, (a·b)·c=a·(b·c).
  (c) For every a∈G, there exists e∈G such that e·a=a·e=a.
  (d) For every a∈G, there exists an element $a\;\cdot \;a^{-1}=a^{-1}\;\cdot \;a=e$.
  (e) If a·b=b·a for all a,b∈G, the group is considered an *Abelian* group.
2. Field: A field is an extension of a group that has two primary operations: addition and multiplication. Both addition and multiplication within a field are Abelian groups, where the operations are commutative and associative. Further, both groups have an identity where e·a=a·e=a, and an inverse where $a\;\cdot \;a^{-1}=a^{-1}\;\cdot \;a=e$. For a∈F:
  (a) Additive Identity: a+0=a
  (b) Additive Inverse: a+(−a)=0
  (c) Multiplicative Identity: a·1=a
  (d) Multiplicative Inverse: $a\;\cdot \;a^{-1}=1$
  Furthermore, a *Finite* Field $F_{q}$ is a field with *q* elements, and exists only if *q* is prime. An extension of this, a *Number* field, is a vector with a finite dimension over rational numbers, often denoted Q. Finally, a *Cyclotomic* Field is a field that is generated by combining a complex root of unity with Q.
3. Ring: A Ring *R* shares similar properties with a Field, and has two binary operations: addition and multiplication. Unlike a field, however, only the addition is an Abelian group. Multiplication is a semigroup, in which it is associative, but it may or may not be commutative. Not all elements have a multiplicative inverse.
4. Homomorphism: A relation between two algebraic structures, such as a Group, Field, or Ring, that preserves the structure. A homomorphism does not have to be bijective, which means that each element in set *A* does not necessarily map to a single element in set *B*.
5. Isomorphism: A function between two algebraic sets where each element in set two is the image of exactly one element from set one, that is also structure preserving. It is an extension of a homomorphism that must be bijective.
6. Automorphism: An Automorphism further extends an Isomorphism as the domain and codomain are of the same structure. In other words, an Automorphism is a bijective function that maps a structure onto itself while simultaneously preserving its operations. For example, complex conjugation preserves addition and multiplication, is bijective, and maps its output to itself.

### 2.2. Homomorphic Basics

Homomorphic encryption, as a generalization, should support, at a minimum, the following functions:

1. Encode: Convert a plaintext set to a polynomial form. The way this function is handled differs significantly between FHE algorithms. For example, CKKS uses a complex canonical embedding map, while data in BGV is simply placed from a slot into a coefficient.
2. KeyGen: Using a distribution that is dependent on the scheme chosen, generate a secret key (SK) and public key (PK).
3. Encrypt: Using the PK, encrypt the data. Similar to KeyGen, this function is dependent on the FHE algorithm.
4. Evaluate: Different evaluation functions are supported by different algorithms. For example, TFHE supports Boolean operation, and most HE algorithms support addition and multiplication as a bare minimum.
5. Decrypt: Using the SK, output the encoded message. Considering that the noise did not grow too large during the evaluation process, the message should be recoverable.
6. Decode: Convert the plaintext from a polynomial back into a list of numbers. If the scheme used is CKKS, these include complex floating point numbers.

In this work, our aim is to support two primary variants of FHE: Homomorphic Encryption for Arithmetic of Approximate Numbers (HEANN) [10], also known as CKKS, and Brakerski–Gentry–Vaikuntanathan (BGV) [7]. The two algorithms differ in the types of numbers supported:

- BGV focuses on integer arithmetic, supporting exact computations on the encrypted integer values. Noise reduction is done via modulus switching.
- CKKS is a method that works on floating point and complex numbers. Plaintext is multiplied by a scaling factor, and the imaginary portion of the plaintext is encoded via the canonical embedding process. Noise is managed via rescaling.

### 2.3. Number Theoretic Transform

As mentioned previously, one of the main limitations of HE is the reliance on expansive polynomials, spanning up to hundreds of thousands of coefficients. This is especially an issue during polynomial multiplication. We illustrate the simpler version of polynomial multiplication, a linear convolution between *g* and *h* in Equation (1). This equation illustrates the schoolbook method, and has a time complexity of $O(n^{2}).$

(1) $$y\left[k\right]=\left(g*h\right)\left[k\right]=\sum_{i=0}^{k}g_{i}h_{k-1}$$

However, multiplication over polynomial rings in the form of $Z_{q}\left[x\right]/\left(x^{n}+1\right)$, as is the case with popular FHE cryptosystems, relies on negacyclic convolution, rather than linear convolution. The negacyclic convolution, also known as a negative wrapped convolution, is defined in Equation (2).

(2) $$\sum_{k=0}^{n-1}x^{k}\left(\sum_{i=0}^{n-1}g_{i}h_{k-i}-\sum_{i=k+1}^{n-1}g_{i}h_{k+n-i}\right)\;\mathrm{mod}\;\left(q\right)$$

As multiplications are a common operation in FHE, the time complexity must be reduced. A common approach for handling convolutions is the fast Fourier transform (FFT); however, FHE operates on an integer ring. The NTT offers an integer-based equivalent that can transform a polynomial into a frequency-domain equivalent [17]. Unlike the FFT, which operates in the frequency domain, the NTT does not have a physical meaning. Despite this, the NTT still supports the convolution theorem. Both the NTT and its inverse, the Inverse Number Theoretic Transform (INTT), are based on the primitive *n*-th root of unity, ω, for linear convolutions. The NTT of a polynomial coefficient $a_{j}$ is described in Equation (3), and its inverse in Equation (4).

(3) $$\begin{array}{cc} NTT(a_{j}) & =\sum_{i=0}^{n-1}\omega^{ij}a_{i}\;\mathrm{mod}\;q\\ j & =0,1\ldots ,n-1 \end{array}$$

(4) $$\begin{array}{cc} a_{i}=INTT\left({\hat{a}}_{i}\right) & =n^{-1}\sum_{i=0}^{n-1}\omega^{ij}{\hat{a}}_{i}\;\mathrm{mod}\;q\\ j & =0,1\ldots ,n-1 \end{array}$$

The primary difference between the NTT for a linear convolution and a negacyclic convolution is that the negacyclic relies instead on the 2n-th root of unity, ψ, rather than ω. We define the NTT and INTT for negacyclic convolutions in Equations (5) and (6).

(5) $$\begin{array}{cc} NTT(a_{j}) & =\sum_{i=0}^{n-1}\psi^{2ij+i}a_{i}\;\mathrm{mod}\;q\\ j & =0,1\ldots ,n-1 \end{array}$$

(6) $$\begin{array}{cc} a_{i}=INTT\left({\hat{a}}_{i}\right) & =n^{-1}\sum_{i=0}^{n-1}\psi^{2ij+j}{\hat{a}}_{i}\;\mathrm{mod}\;q\\ j & =0,1\ldots ,n-1 \end{array}$$

It is clear from Equations (3) and (4) that the NTT is of time complexity $O(n^{2})$. There exists other, faster alternatives, such as the Cooley–Tukey NTT variant borrowed from the FFT [18], reducing the time complexity to O(nlogn). There is also the Gentleman-Sande for a fast INTT variant [19]. Further, the NTT is not a convolution itself: it only serves to reduce the time complexity of convolutions, whether they be linear, cyclic, or negacyclic. Linear operations, such as addition and multiplication, can all be performed in the NTT domain, often referred to as evaluation form. Both multiplication and addition in this evaluation representation are component wise, reducing the complexity of convolutions down to O(n). For the NTT representation of polynomials $\hat{A}$ and $\hat{B}$ composed of coefficients $\hat{a_{i}}$ and $\hat{b_{i}}$, their sum is denoted in Equation (7), and their product in Equation (8).

(7) $$\hat{A}+\hat{B}=\sum_{i=0}^{n-1}\hat{a_{i}}+\hat{b_{i}}\;\mathrm{mod}\;q$$

(8) $$\hat{A}*\hat{B}=\sum_{i=0}^{n-1}\hat{a_{i}}\hat{b_{i}}\;\mathrm{mod}\;q$$

Accelerating the NTT for FHE has been a well-researched topic, as are other lattice-based cryptosystems [20,21,22]. For example, Ye et al. [23] offers a parameterized solution for the NTT on an FPGA, enabling various polynomial degrees and moduli, showcasing up to a 4.3× speedup over state-of-the-art implementations. Their work specifically targets lattice-based cryptography, such as FHE. Another example, NTTGen, is a NTT generator for general FPGA applications. They offer a customizable pipeline, which allows for user-defined latency and throughput [23].

Although the acceleration of NTT can have a significant speedup on FHE applications, we believe that the topic is to be thoroughly explored. Therefore, we do not implement the NTT within DARTPHROG. NTT implementations show that it can be resource-hungry, utilizing over 75 k Look-Up Tables (LUTs) and 61 k FlipFlops (FFs) and 700 KB of Block Ram (BRAM) in an instance of the work by Kurniawan et al. [24], all while limiting the polynomial degree. Instead, we aim to evaluate the potential performance of an FPGA when solely dedicated to accelerating FHE functions while the polynomial is in evaluation form. For this reason, we assume that all ciphertexts and plaintexts loaded into DARTPHROG via DMA are already in evaluation form.

### 2.4. Transforming Coefficients

As previously described, the coefficients for a ciphertext must be adequate enough to encompass both the base-noise level, and the anticipated noise growth from homomorphic operations such as addition and multiplication, without interfering with the encrypted data. A larger coefficient in the ring $Z_{Q}$ is directly proportional to a deeper circuit depth. This results in FHE systems seeing coefficients as large as $2^{10}$ bits. Unfortunately, arithmetic with integers of that scale requires significant resources and is more computationally expensive than typical 32-bit and 64-bit word operations.

Breaking these large integers into small integers for addition is trivial; however, multiplication is significantly more complex. An answer to this is the Karatsuba algorithm, which is a fast multiplication algorithm published in 1962 [25]. The Karatsuba algorithm reduces a multiplication of two *n*-digit numbers into three multiplications to n/2-digit numbers. Through cascading Karatsuba multiplications, an *n*-digit multiplication can be reduced to a maximum of $n^{log_{2}\left(3\right)}$ single-digit multiplications.

There have been multiple attempts at implementing Karatsuba multiplication in FPGAs, with various findings. Flex Karatsuba (FlexKA), for example, found that their Karatsuba FPGA implementation only began outperforming schoolbook multiplication at vector sizes greater than 1024 bits [26]. Furthermore, another work by Heidarpur et al., [27] demonstrates that an overlap-free karatsuba variant has a relatively high gate count. A 400+ bit operand would require more than 100 k gates, and a 256-bit operand would require approximately 50 k gates. While Karatsuba may be a strong contender for integers greater than 1kb, this is out of the range of conventional FHE.

Instead, we look towards the Residue Number Systems (RNSs). An RNS takes a large integer, *Q*, and breaks it down into small, word-size integers, referred to as the moduli group. Each moduli within an RNS must be coprime, that is, the greatest common factor between two moduli in an RNS must be 1. In the RNS domain, a large integer *Q* is represented as $Q_{L}=\left\{Qmod\left(q_{0}\right),Qmod\left(q_{1}\right),\ldots ,Qmod\left(q_{L-1}\right)\right\}$ where each residue is denoted as $q_{i}$. The largest value of *Q* that can be represented by the RNS is therefore defined as $\prod_{i=0}^{L-1}q_{i}$. Operations on two large integers *P* and *Q* can be calculated component wise, given that *P* and *Q* are represented in the same RNS domain.

The original *Q* can be recovered via the Chinese remainder theorem (CRT). However, since all arithmetic supported by DARTPHROG can be performed in the RNS domain, we do not implement a CRT solver and instead defer that responsibility to the host processor. Each of the major FHE variants covers RNS implementations, including CKKS [28] and BFV [29].

Although this is a simple system for breaking large coefficients down, the complexity in hardware is directly derived from the complexity of the Modular Reduction algorithm used. A recent publication, dubbed the Hardware Optimized Modular-Reduction (HOM-R) system, is the ideal candidate for our purposes, as it targets arbitrary bases for Modular Reduction on an FPGA in an efficient manner [16]. The HOM-R system is an improvement in hardware over conventional Modular Reduction approaches, such as the Montgomery [30] or Barrett [31] algorithms, as it does not require any complex primitives, such as Digital Signal Processing (DSP) slices. Furthermore, the HOM-R system does not require an integer conversion, as does Montgomery Reduction. This allows the HOM-R system to be seamlessly integrated into an Advanced eXtensible Interface (AXI) Stream bus for multiple parallel reducers with minimal impact on the place-and-route tools for the FPGA, as well as seamless integration into an ALU.

### 2.5. Generalized Homomorphic Evaluation

Within this subsection, we cover homomorphic operations supported by DARTPHROG, which includes addition and multiplication between two ciphertexts, as well as addition and multiplication between a ciphertext and a plaintext. Specifically, Equations (9)–(14) define the underlying supported mathematical operators in DARTPHROG. We do not support operations that require the NTT and INTT, such as bootstrapping and relinearization. We instead defer those responsibilities to the host system. Further, we do not discuss the encoding, encryption, or decryption of any of the support cryptosystems, as those operations are to be performed by the host system.

For all operations discussed within this section, polynomials are expected to be in the RNS evaluation form, in the representation (c1,c0). We describe each evaluation method for ciphertexts $C^{a}$ and $C^{b}$, both of which are in the same modulus $q_{l}$. For CKKS acceleration, $C^{a}$ and $C^{b}$ must be of the same level Δ. We also describe operations with plaintext *P*, which is in the same modulus $q_{l}$ as $C^{a}$ and $C^{b}$. The addition between two ciphertexts is displayed in Equation (9), and addition between a ciphertext and plaintext is shown in Equation (10). These are simple operations between polynomials, and only results in linear noise grown in the case of ciphertext/ciphertext addition.

(9) $$EvalAdd\left(C^{a},C^{b}\right)=\left({\left[C_{0}^{a}+C_{0}^{b}\right]}_{ql},{\left[C_{1}^{a}+C_{1}^{b}\right]}_{ql}\right)$$

(10) $$Add\left(C^{a},P\right)=\left({\left[C_{0}^{a}+P\right]}_{ql},{\left[C_{1}^{a}\right]}_{ql}\right)$$

To introduce subtraction into the system, we must keep the difference between two numbers within the ring $Q_{l}$. In the software, we can support the Modular Reduction of negative numbers. With the HOM-R system used in this paper, we can operate only on positive integers. To ensure that we do not try to reduce a negative number with HOM-R, we add the additive inverse of the subtrahend to the minuend, avoiding the subtraction operation. The negative inverse of an integer *a* in modulo ring $q_{l}$, denoted *b*, is defined as $a+b\;\mathrm{mod}\;q_{l}\equiv 0$. If we guarantee $0\leq a<q_{l}$, then the additive inverse of $a\;\mathrm{mod}\;q_{l}$ is simply $q_{l}-a$. The full subtraction is therefore defined in Equation (11) for ciphertexts, and Equation (12) for plaintexts.

(11) $$EvalSub\left(C^{a},C^{b}\right)=\left({\left[C_{0}^{a}+\left(q_{l}-C_{0}^{b}\right)\right]}_{ql},{\left[C_{1}^{a}+\left(q_{l}-C_{1}^{b}\right)\right]}_{ql}\right)$$

(12) $$Sub\left(C^{a},P\right)=\left({\left[C_{0}^{a}+\left(q_{l}-P\right)\right]}_{ql},{\left[C_{1}^{a}\right]}_{ql}\right)$$

Unlike simple additions and subtractions, multiplication on encrypted data in FHE is more complex. Ciphertext/ciphertext multiplications, shown in Equation (13), can be evaluated as the polynomial multiplication of the input ciphertexts. This effectively increases the degree of the cipher product. While this increase in degree will make further computations more expensive, the output product can be relinearized back down to two components, c0 and c1, through relinearization. Relinearization differs between the three schemes that we seek to support, so we defer this responsibility to the host machine. Therefore, any products resulting from ciphertext/ciphertext multiplications in DARTPHROG should be offloaded to the host processor, relinearized, and reloaded back into DARTPHROG.

(13) $$\begin{array}{cc} EvalMult(C^{a},C^{b})= & \left(d_{0},d_{1},d_{2}\right)\\ = & ({\left[C_{0}^{a}\;\cdot \;C_{0}^{b}\right]}_{ql},\\ \; & {\left[C_{0}^{a}\;\cdot \;C_{1}^{b}+C_{1}^{a}\;\cdot \;C_{0}^{b}\right]}_{ql},\\ \; & {\left[C_{1}^{a}\;\cdot \;C_{1}^{b}\right]}_{ql}) \end{array}$$

Because ciphertext/ciphertext multiplications are computationally expensive, one should aim to use ciphertext/plaintext multiplications when possible. Each factor in a multiplication must be carefully evaluated to determine whether it must be encrypted or not. Only client data that must be preserved should remain encrypted; data only available to the host system, and not the client, can remain as plaintext. As shown in Equation (14), ciphertext/plaintext multiplications do not increase the degree in the resulting product, allowing further computations to take place within DARTPHROG without having to reload the data into the host.

(14) $$Mult\left(C^{a},P\right)=\left({\left[C_{0}^{a}\;\cdot \;P\right]}_{ql},{\left[C_{1}^{a}\;\cdot \;P\right]}_{ql}\right)$$

### 2.6. FHE Acceleration

Since FHE is significantly slower than other non-privacy-preserving cryptographic methods, it is an obvious target for acceleration. There are multiple efforts to accelerate FHE, ranging from software, to Graphical Processing Units (GPUs), to configurable hardware like FPGAs, to Application-Specific Integrated Circuit (ASICs) [32].

Software acceleration methods on a conventional CPU are an interesting choice, as CPUs are not designed for massive parallelism. However, their flexibility for general-purpose programming can potentially facilitate fast implementations and experimentations, as well as creative approaches to FHE. One commonality between different software approaches is to utilize the Intel Advanced Vector Extensions 512 instruction set (AVX512), with means to support the large coefficients required for HE. For example, Boemer et al. [33] designed a software library that accelerates modular multiplication and the NTT operation. They optimize loops by unrolling them and operating on the data in parallel, at least as much as the AVX512 ISA and CPU architecture will allow.

Other CPU acceleration methods look at single, expensive functions within HE, such as the trace-type function in CKKS. This function is performed by repeating homomorphic rotations followed by additions. Ishimaki et al. proposed another loop-unrolling method, reducing the number of expensive operations by relying on the properties of automorphisms and multicore processing [34].

GPUs offer more configurability than general purpose processors, and they offer wide data buses that can be utilized for the large arithmetic required of FHE. A work with goals similar to those of DARTPHROG, but implemented on a GPU, is that of Shivdikar et al. [35], who proposed an accelerator for polynomial multiplication. They specifically targeted memory accesses within the GPU increase throughput. Another work on GPUs by Shen et al. [36] aimed at optimizing BGV, BFV, and CKKS by combining previous acceleration methods. Their implementation also targeted two different NTT methods, exploring their impact on memory limitations. Moreover, GPUs offer the capability to distribute intensive loads across multiple cards. For example, Lupascu and Lei et al. offer two different approaches for acceleration based on load balancing [37,38].

The next level of design customization following GPUs are FPGAs, which DARTPHROG implements. One of the first FHE accelerators to implement bootstrapping on an FPGA, F1, which served as inspiration for many of the following accelerators. F1, similar to DP, has both a physical hardware implementation and a custom Instruction Set Architecture (ISA). Where DP seeks to simplify the ISA, F1 sought to give more control to the user. For Modular Reduction, F1 relied on optimized Montgomery multipliers [39].

FAB, introduced by Agrawal et al., [39] proposes a full FHE accelerator that includes basic homomorphic primitives and bootstrapping. FAB, similar to the GPU works by Lupascu and Lei, allows for collaborative efforts between multiple cards via a CMAC core on the FPGA. Unlike DARTPHROG, FAB relies on storing polynomial data in Ultra RAM (URAM), thereby limiting the depth and coefficient size of the FHE implementation.

Poseidon, by Yang et al., [40] implements another FHE accelerator that relies on the unique properties of automorphisms to allow for resource reuse. This strategic recycling strategy allows for larger computational units, as their resources can be shared between operations. Modular Reduction is handled via the Barrett reduction for multiplications, and simple subtraction in the case of additions.

Another accelerator, called the FHE Accelerator for Scalable-parallelism with Tunable-bit (FAST), seeks to improve upon the time required specifically for bootstrapping within FHE. FAST is the first FPGA accelerator to implement the Advanced Bootstrapping Algorithm (ABA), which relies on lower memory costs to accelerate bootstrapping [41]. Their performance, utilizing ABA shows an average 2x increase in throughput over FAB and Poseidon.

## 3. System Architecture

As DP is a full system, spanning both an FPGA and host integration, custom software development is required for FPGA deployments. Both software and hardware developments are described within this section.

**Figure 2:**
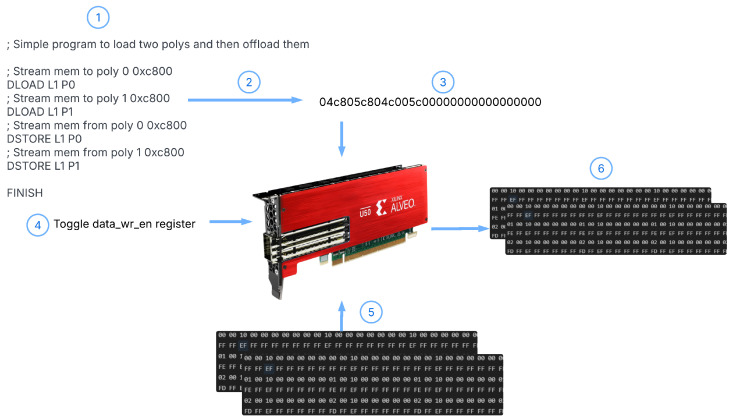
A flowchart of the procedure for operating on polynomials with DARTPHROG.

The complete flowchart for operating on polynomials using DARTPHROG is shown in Figure 2. The supporting architecture for these steps is further defined within this section. The steps in the flowchart are described as follows:

1. The assembly code is written by the developer.
2. The linter writes the binary control values based on the assembly.
3. The binary values are then streamed into the Alveo card.
4. data_wr_en is enabled. All subsequent data streamed to the card will be streamed to the processor, rather than instruction memory.
5. The polynomials are streamed in order according to the microcode. In this example, Polynomial 0 is streamed first, while Polynomial 1 is streamed second.
6. The processed polynomials are finally streamed out of the card.

Register writing is performed manually using the register access tool bundled with the Xilinx Direct Memory Access (XDMA) drivers, but could just as well be automated. Data are also streamed in via the small C programs bundled with the Xilinx XDMA drivers [42].

### 3.1. Instruction Set Architecture

FHE is composed of large operations, such as polynomial multiplications and additions that span hundreds to thousands of coefficients. Writing microcode for each coefficient would not only be tedious, but also would limit throughput as the scale of the microcode would grow with each polynomial operation. Therefore, we opted to formulate our own DARTPHROG Instruction Set Architecture (DISA) that can support operations essential to FHE, where each instruction describes the operation on an entire ciphertext, which is composed of two polynomials, or a plaintext, which is composed of a single polynomial. This microcode is loosely based on THUMB, as we have a 16-bit instruction set and eight registers. DISA is more limited in scope, as we are restricted by operations available to FHE algorithms in evaluation mode. Our ISA is summarized in Table 1.

The first two instructions in the table are LD (load) and ST (store) operations, which describe the Direct Memory Access (DMA) functionality on the card. A load LD dictates that data be loaded to the card from the host system. In the case that the Homomorphic Program loaded to DARTPHROG is waiting for a load before proceeding, and in the case of a data hazard that relies on Rd, a ready bit in the Peripheral Component Interconnect Express (PCIe) register space will be asserted, indicating that the card is ready for a new ciphertext or plaintext to be loaded into Rd. This will allow the programmer to load data to the card, perform some *n* operations, and continue. Similarly, as all loads do not have to take place at the start of a homomorphic program, all stores do not have to take place at the end of a program. A user can load and store data throughout the runtime of the loaded program.

The stored data are expected to be in evaluation form, meaning that it has been converted to the frequency domain via the NTT, and reduced to a series of residues in a Residue Number System (RNS). The RNS requirement is described further in the next subsection.

A parameter unique to the DISA LD/ST operation is the polynomial level (PL), in bits 4 and 5. The PL is used within DARTPHROG to dictate how data is handled, as additions and multiplications between ciphertexts and plaintexts are computed differently depending on the operands. A PL can have three values:

- 2’b00: Plaintext;
- 2’b01: Ciphertext of level one, composed of (c0,c1);
- 2’b10: Ciphertext of level two, composed of (d0∗,d1∗,d2∗).

The arithmetic operations, ADD, SUB, and MUL, all depend on the level of the ciphertext to operate properly. The control signals from the control unit apply the appropriate masking, discussed later in this section, depending on the level of the ciphertext. The level of the ciphertext is also tracked within DARTPHROG. For example, the multiplication between two level one ciphers results in a product with one level two cipher. The current level of each cipher can be read via the PCIe bus register access.

To assist with programming DARTPHROG, we implemented an assembler in Python 3. This assembler allows us to generate machine language for the accelerate compliant with Table 1. A small, sample program is shown in the following code snippet.


; Stream mem to poly 0 and 1
DLOAD L1 P0
DLOAD L1 P1
; P2 = P0 + P1
ADD P0 P1 P2
DSTORE L1 P2
FINISH
        

### 3.2. Hardware Implementation

**Figure 3:**
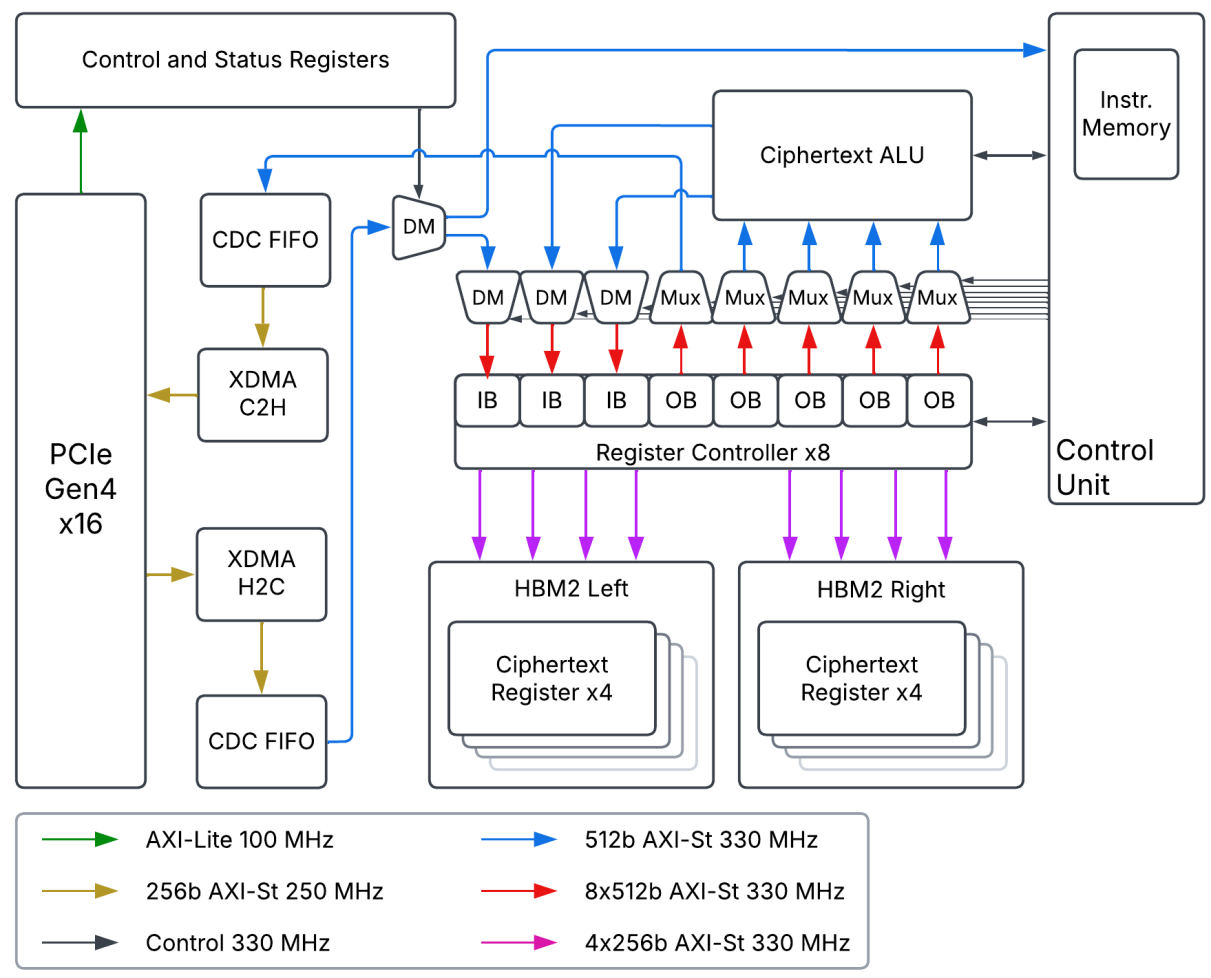
The DARTPHROG architecture. CDC FIFO: clock domain crossing first-in first-out buffer. XDMA: Xilinx DMA. C2H: card to host. H2C: host to card. DM: demultiplexer. Mux: multiplexer. ALU: arithmetic logic unit. HBM2: high bandwidth memory 2. IB: input buffer. OB: output buffer.

The hardware architecture, shown in Figure 3, can be separated into four primary components: the XDMA subsystem, the register bank, the ALU, and the control unit.

The DARTPHROG architecture design was guided by a desire to increase the parallelism offered by hardware such as an FPGA. While we could conceivably accelerate a single mathematical primitive, such as an addition or multiplication, we wanted to give the programmer the option to run multiple operations in parallel. This requirement is dictated by the long processing times of ciphertexts, even in evaluation form. For this reason, we opted to make our design superscalar. We discuss this in greater depth further in this section.

However, the first question is how to integrate the accelerator into a common FHE software library such as Microsoft Simple Encrypted Arithmetic Library (SEAL) [43] or OpenFHE [44]. For full-scale processor integration, an isolated FPGA or ASIC, and the control unit design will not suffice. Our answer to this issue is to utilize the Xilinx DMA architecture, XDMA, to integrate our system into a host. This integrates a card-to-host (C2H) engine for offloading data from the FPGA, and a Host-to-Card (H2C) for loading data to the FPGA.

### 3.3. Polynomial Registers

Our ciphertext data is stored in “registers” within the HBM. Other designs, such as F1 and FAB, utilized on-chip memory for polynomial storage during operations. However, since we are limited due to the size of the U50 (compared to the U280 in FAB and F1), we must prioritize sparing memory where necessary. Each register spans four pseudochannels of HBM, with each channel is 256 bits, allowing for massively parallel reads and writes. Storing the polynomials this way has a few perks. First, the HBM has a theoretical bandwidth of 316 GB/s, but is power constrained to roughly 200 GB/s. Spreading across eight registers allows for 25 GB/s, or 200 Gbit/s per register.

Second, the HBM allows for polynomials of variable depth. We offer a programmable register on the PCIe bus for the programmer to declare the degree of the polynomial. As polynomials can span up to 1 kbit per coefficient, and up to 128 k coefficients, we would quickly become limited by on-chip Block Random Access Memory (BRAM) resources. However, by utilizing the HBM, we can support the polynomials of a degree well beyond what is considered possible.

Further, because we do not perform the relinearization on DP, we must be prepared to store an extra term. Recall that a polynomial multiplication results in three terms, d0, d1, and d2, each equivalent in size to one of the terms, c0 or c1. This results in a 50% increase in memory usage when a ciphertext/ciphertext multiplication is performed. Thanks to the depth of our HBM registers, we can easily accommodate this requirement.

Third, due to the 512 bit data bus offered in DP, congestion on the FPGA can begin to accumulate with every new component. This would be especially true if we were to direct all data to on-chip BRAM and URAM resources, as those memory devices are not spaced in a manner that is efficient for handling significant amounts of congestion. The HBM, however, spans the entirety of the south end of the FPGA, allowing the 512 bit data bus to be adequately spread to reduce congestion and improve timing for the design.

**Figure 4:**
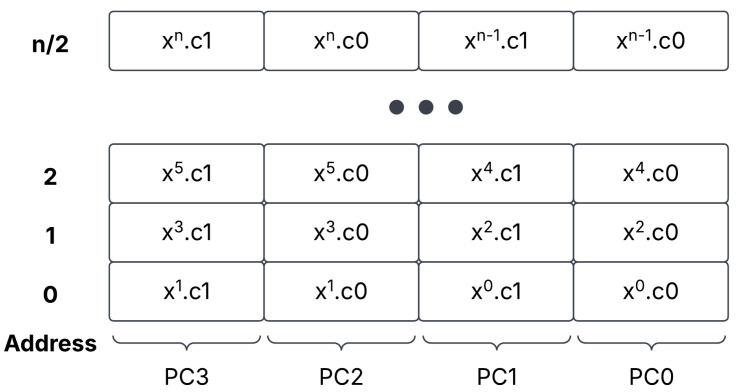
The default ciphertext storage architecture, with c0 and c1 being stored side by size.

**Figure 5:**
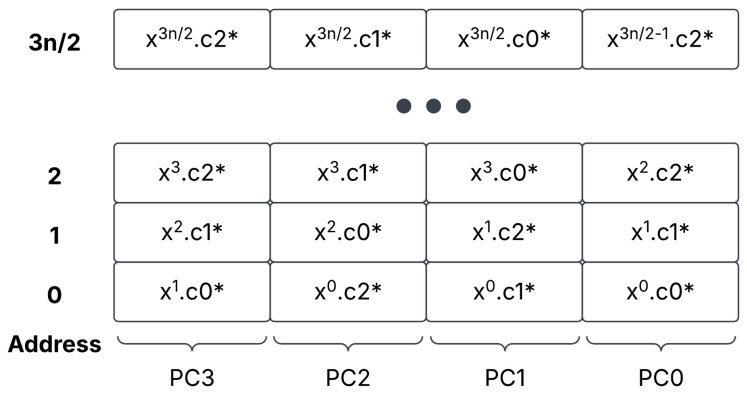
A-level two ciphertext storage in HBM, with the terms d0, d1, and d2 interleaved together. A ’*’ indicates that the ciphertext component is part of a level two polynomial.

To account for ciphertext portions c0 and c1, we use a unique approach to data storage. We store the two halves of a cipher side by side, as in Figure 4. As each polynomial is stored in evaluation form, we can depend on this storage type to assist with speedy execution in the ALU. Next, as mentioned, we must store d0, d1, and d2 after a multiplication of ciphertext. As the ciphertext multiplier outputs d0, d1, and d2 in parallel, we integrate the three coefficients in HBM, which are shown in Figure 5.

**Figure 6:**
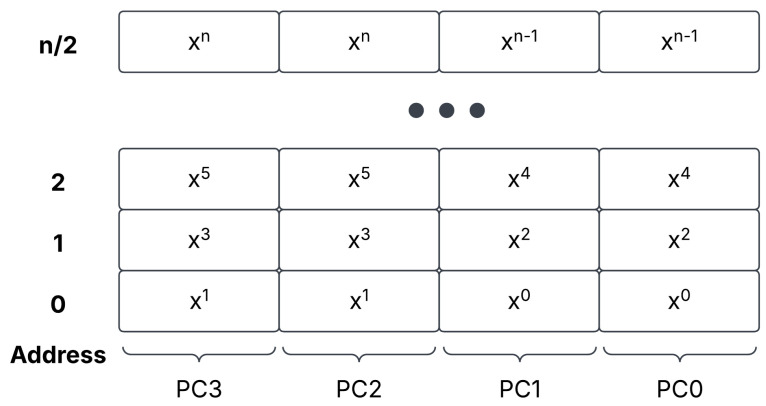
The plaintext storage architecture, with the plaintext duplicated side-by-side in HBM.

Finally, we discuss the storage of plaintexts. As we do not perform relinearization from ciphertext/ciphertext multiplications, we encourage the user to rely on plaintext/ciphertext multiplications when possible. Plaintexts are also stored in polynomial registers. As we will discuss later in the ALU Section 3.6, we duplicate the plaintext input. This is due to the plaintext only being the equivalent size of a single c0 or c1 term. We outline this storage architecture in Figure 6.

### 3.4. Control Unit

The control unit maintains the register and arithmetic scoreboard, as well as monitoring the two read and four write buses. When issuing an instruction, the control unit will mark the respective registers Rn, Rd, and Rs as busy. The control unit will also mark the respective arithmetic unit as busy. For completions, the control unit monitors ready signals from the register bank and arithmetic unit.

Furthermore, the control unit is also responsible for monitoring the polynomial level stored in each register. Initial levels for each polynomial are dictated by the Data Load (DLOAD) instruction, and can only change during a ciphertext/ciphertext multiplication, as this is when (c1,c0) can expand to (d2,d1,d0). In this instance, the Rd register will be upgraded from a level one ciphertext to a level two ciphertext. The control unit exposes the polynomial level to the register bank, so that the bus arbiters know how many words of data to put on the bus in case of a read transaction. Likewise, this is also how the bus arbitrators know how many words of data to write into the HBM in the case of a write transaction.

One final responsibility of the Control Unit is to maintain the instruction memory and program counter. After the instruction memory is streamed in over DMA to the control unit, it is stored in BRAM. The control unit will increment the program counter when a new instruction is issued, and stall in the case of a data or structural hazard.

### 3.5. Hardware Optimized Modular Reduction

Most other accelerators use some form of either Barrett or Montgomery reduction. We were the first to implement the Hardware Optimized Modular Reduction (HOM-R) method. While Barrett and Montgomery may be efficient in software implementations, we argue that HOM-R is ideal for hardware [16]. HOM-R relies on a series of lookup tables to calculate the output product. More importantly is the fact that it can be pipelined without the requirement for DSP slices. This allows the place and route tools to put the HOM-R system anywhere on the FPGA with available Look-Up Tables (LUTs) and Flip Flops (FFs), increasing the maximum operating frequency.

For DP, we utilize compile-time parameters to define the RNS of DP. For our purposes, we utilize eight 32-bit integers that are passed as top-level parameters to the ALU HOM-R units. The individual limbs of (c1,c0) are stored in memory as $q_{7},q_{6},\ldots ,q_{0}$. This allows for a predictable data arrangement so that the output of the arithmetic units can be efficiently reduced with their respective base-defined HOM-R units.

### 3.6. Arithmetic Logic Unit

**Figure 7:**
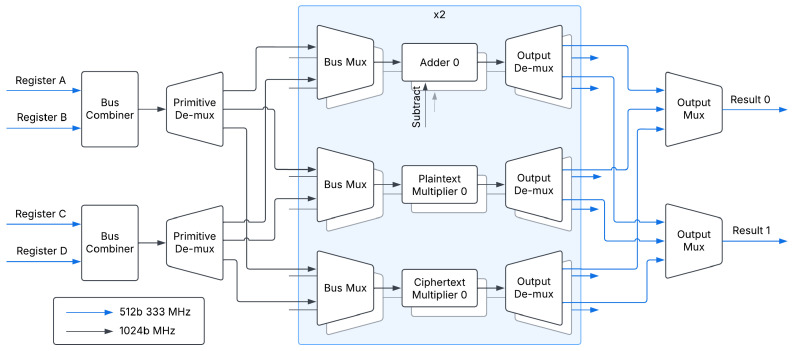
The DARTPHROG arithmetic logic unit, showcasing the two adders, two plaintext multipliers, and two ciphertext multipliers.

The function behind the ALU, shown in Figure 7, is to efficiently operate on streamed data. To allow for a superscalar architecture, there are two input data buses, and two output data buses. Further, each input data bus combines two input streams of data, Rs and Rn from the opcodes of the current instruction. This effectively turns our four 512-bit input buses into two 1024-bit input buses, which are then directed to the correct arithmetic unit via AXI test bits. It is up to the bus arbiter from the register bank to determine the destination of each input stream. In other words, the arbiter ensures that there is no bus contention between the two parallel instructions.

In a similar manner, the output stream of an arithmetic unit is reduced to 512 bits. As we have two possible, active buses, we have two potential output streams. The destination for these streams, Rd, is determined by the control unit.

The ALU contains two copies of three different functions, each of which has a HOM-R unit tied to the output. This is allowed as HOM-R is an efficient Modular Reduction method, and ensures that the output data stays in the ring [0,q). The ALU acts on an AXI-stream basis, where data streamed in are operated on, and then streamed out. It is expected that an input word is in the form of (c1,c0) for ciphertexts, where the upper 256 bits consist of c1, and the lower 256 bits consist of c0. Further, we anticipate plaintexts to be streamed in as (p,p), where the plaintext is duplicated in the upper and lower 256 bits.

The first unit is the adder, which can be turned into a subtractor via a control bit. Recall from Section 2 that we must keep all data greater than zero if we are to properly utilize the HOM-R unit. In order to accomplish this with subtractions, we calculate the additive inverse using Equation (12). The “subtract” control bit from the control unit will enable this function; otherwise, data simply passes through the inverter untouched. Only Rn is passed through the inverter, as we do not implement a function for a double negative addition that would invert Rn. We then add the two input streams together before reducing them with HOM-R.

Further, recall from the previous subsection that we will have two copies of the plaintext directed to the input stream to be added to the 512-bit ciphertext (c1,c0) word. This would effectively calculate (c1+p,c0+p); however, this is incorrect for a ciphertext/plaintext addition. In order to retrieve the correct result (c1,c0+p), we mask the upper half of the plaintext word, thereby calculating (c1+0,c0+p).

Plaintext multiplication is the simplest of the four arithmetic operations that DP supports. Recall that a ciphertext multiplied by a plaintext would be (c1∗p,c0∗p). Since plaintext is already doubled in the word that is streamed into the ALU, we do a simple component wise multiplication in the case of a plaintext/ciphertext multiplication.

On the other hand, ciphertext/ciphertext multiplication is the most complicated operation supported by DP. Recall from Equation (13) that, from (c0,c1), we retrieve three components, (d0,d1,d2), of which d0 and d2 are produced through multiplications of $c0\;\cdot \;c0^{\prime }$ and $c1\;\cdot \;c1^{\prime }$, respectively. d1, however, is the sum of $c0\;\cdot \;c^{\prime }$ and $c1\;\cdot \;c0^{\prime }$, requiring three operations. For this function, we rely on lazy Modular Reduction and only reduce the output of d1 after the $c0\;\cdot \;c1^{\prime }+c1\;\cdot \;c0^{\prime }$ operation in order to reduce the latency of the ciphertext/ciphertext multiplication.

### 3.7. Profiler

Homomorphic operations are so notoriously lengthy that popular papers, such as BFV, are entitled Somewhat Practical Fully Homomorphic Encryption [9]. For this reason, efficiency is at the forefront of homomorphic research. We chose to implement four parallel, register controlled, profilers into DARTPHROG. Each profiler works independently and has a runtime programmable start and stop program counter index.

When the instruction at the start index is issued to the read/write bus arbiters, the profiler starts an accumulator that increments every 3.3 ns clock cycle. The profiler will then monitor the scoreboard and check the completion of the respective Rs, Rn, and Rd registers, along with the freeing of the arithmetic unit. The result of the accumulator is then output to a register that can be accessed over PCIe. In this manner, instructions can be monitored individually, as a group, or even as a holistic measurement of the program. The runtime for each profiler can be calculated by multiplying the value in the accumulator by the clock period of 3.3 ns.

## 4. Results

**Figure 8:**
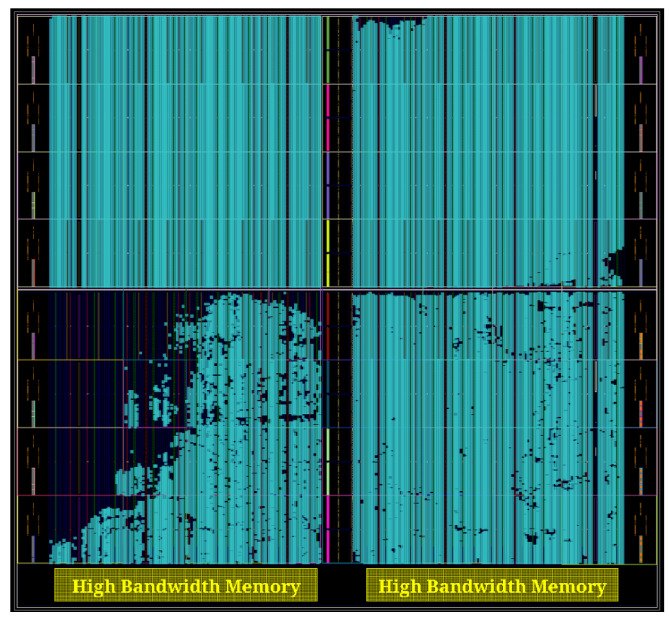
The logic utilization and floor planning of DARTPHROG on the Alveo U50 FPGA. Blue blocks indicate utilized resources, such as LUTs and memory blocks, while unshaded blocks indicate available resources.

DARTPHROG was designed for a Xilinx Alveo U50 data center accelerator card. While this is considered a large device relative to all available FPGAs, it is a compact accelerator compared to the Alveo U280 that is used with most other HE accelerators. In order to meet timing on the U50, we have multiple stages between logic units that implement automatic pipelining to both reduce the net delays and optimize for minimum latency between logic stages. This results in a broad area coverage, as shown in Figure 8.

### 4.1. HOM-R

We present the resource cost for our proposed architecture in Table 2 as the number of resources used, and in Table 3 as the percentage of resources used on the Alveo FPGA. For the ALU arithmetic primitives, our table lists the resource usage per element. Within the ALU, there are two of each element to allow for the parallel operation of each unit. Therefore, the total use between the two arithmetic units within the ALU are double what is presented in the table. These results also include the resource utilization from HOM-R, as the Modular Reduction function is included in each individual unit.

We further present those results for the individual HOM-R units separately in Table 4. For each arithmetic unit, we have two rows. The first row is the total usage required for all HOM-R units within an arithmetic function. For the Adder and PMult units, this is equal to 16 total HOM-R units. Sixteen units are used as each result ciphertext is composed of two polynomials, (c1,c0), and each polynomial coefficient has eight limbs in the RNS domain, resulting in 16 total reductions per operation. However, the Cmult unit has three polynomials per result, (d2,d1,d0). This results in 24 total HOM-R units.

Furthermore, the input width for each HOM-R unit is different between the three functions. In the case of the adder unit, we only have a potential maximum of $log_{2}\left(q_{l}\right)+1$ bits, as an addition only results in a bit-width increase of a single bit, regardless of the input widths. For our 32-bit limbs in DP, this results in a 33-bit number reduction down to a 32-bit base.

Alternatively, multiplication results in an output vector that is equal to the sum of the bit width of the operands, a maximum of $log_{2}\left(q_{l}\right)+log_{2}\left(q_{l}^{\prime }\right)$ bits. Therefore, the input to the PMult HOM-R operator is 64 bits, as each input is a limb of 32-bits.

These two bit-width principles are combined in the case of the PMult operator, which adds two products together. This results in a HOM-R input bit-width of $log_{2}\left(q_{l}\right)+log_{2}\left(q_{l}^{\prime }\right)+1$, or 65 bits, which is the largest of the three arithmetic units.

### 4.2. Functional Unit Results

**Figure 9:**
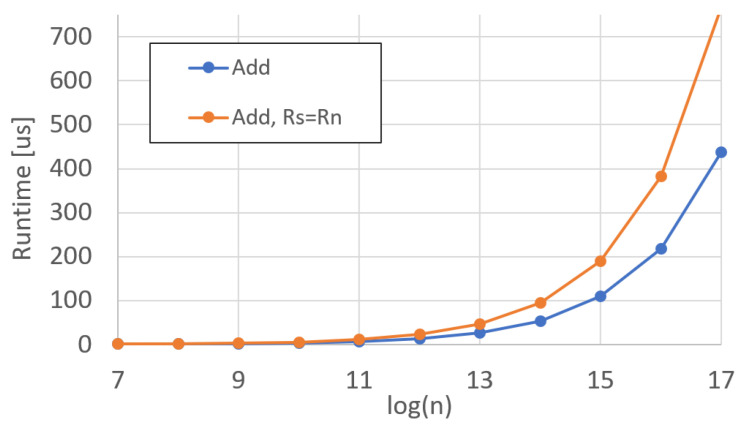
DARTPHROG adder/subtractor runtime.

In order to measure the results from each functional unit, we wrote microcode to load and stored the respective registers while performing the operation under test in between the loads and stores. We measured each operation runtime with the DP Profilers. We performed each test twice, with each test comprising two functional units under test operating in parallel. The results in Figure 9 reflect the average of each addition/subtraction per polynomial degree *n*.

For the adder unit in Figure 9, two different variations of the operations were performed. The first is a simple $R_{d}=R_{s}+R_{n}$, where the two source registers are different from the destination register. The second variant is more complex, where $R_{d}=R_{n}+R_{d}$, requiring a polynomial to be read and written simultaneously.

**Figure 10:**
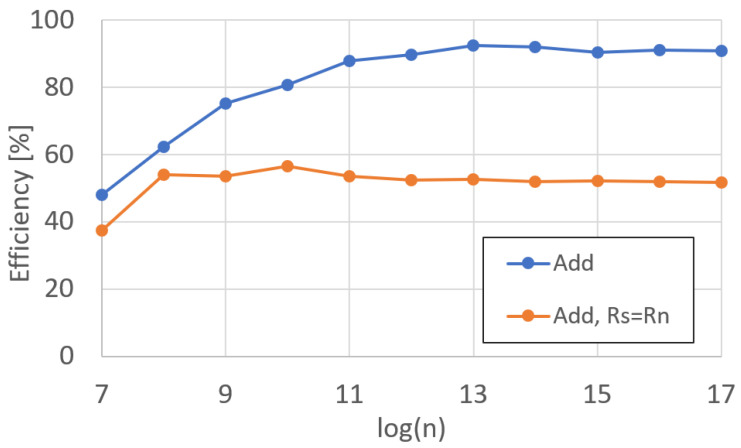
DARTPHROG adder/subtractor efficiency.

Further, we calculate the efficiency of each operation, where maximum efficiency is equivalent to an entire 512-bit addition (256-bit c1 and 256-bit c0) calculation per clock cycle. Variations in efficiency arise from refresh cycles and other delays inherent in the proper functioning of the HBM. The efficiency for addition operations are shown in Figure 10.

The results from the adder unit are also the results of the subtractor, since they are the same functional unit. A control bit from the control unit will invert $R_{n}$, if asserted, effectively making the adder a subtractor. In the case that the control bit is not asserted, the data still passes through the inverter with the same amount of latency as if the inverter were active.

**Figure 11:**
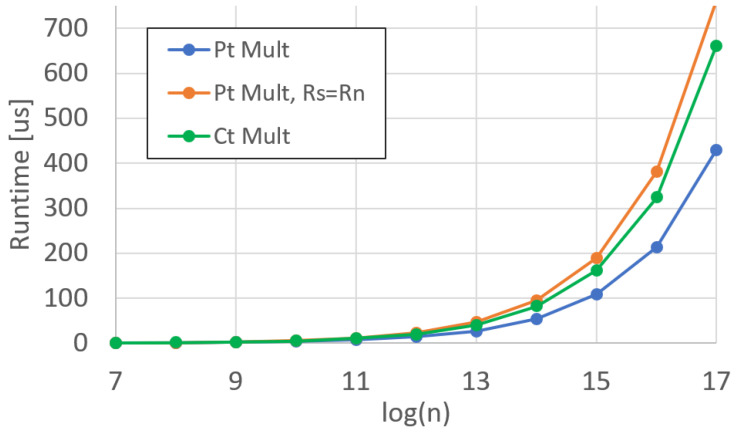
DARTPHROG multiplier runtime.

**Figure 12:**
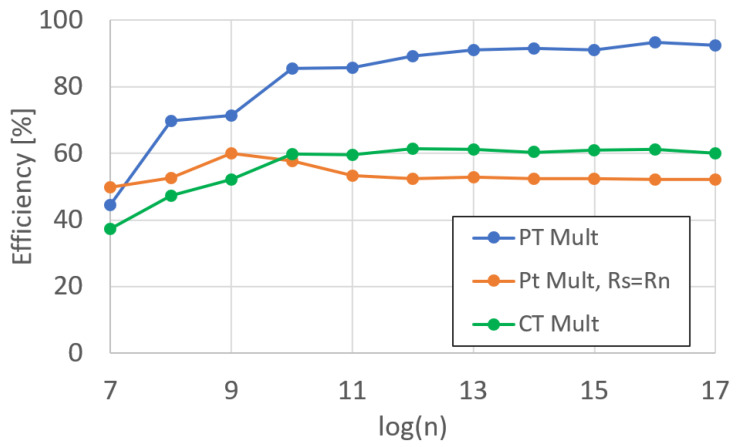
DARTPHROG multiplier efficiency.

Similarly to the adder functional unit, we measured the runtime and efficiency for the PMult and CMult units, shown in Figure 11 and Figure 12, respectively. For Pmult, we show the difference between $R_{d}=R_{s}\;\cdot \;R_{n}$ and $R_{d}=R_{n}\;\cdot \;R_{d}$.

For CMult, we are only able to perform $R_{d}=R_{s}\;\cdot \;R_{n}$, as the CMult product results in three terms, as opposed to two. This is equivalent to a 50% increase in memory requirements. If we were to write to the same register that we were reading from, we would overwrite the data in the register before the product could be calculated, resulting in an incorrect multiplication.

**Figure 13:**
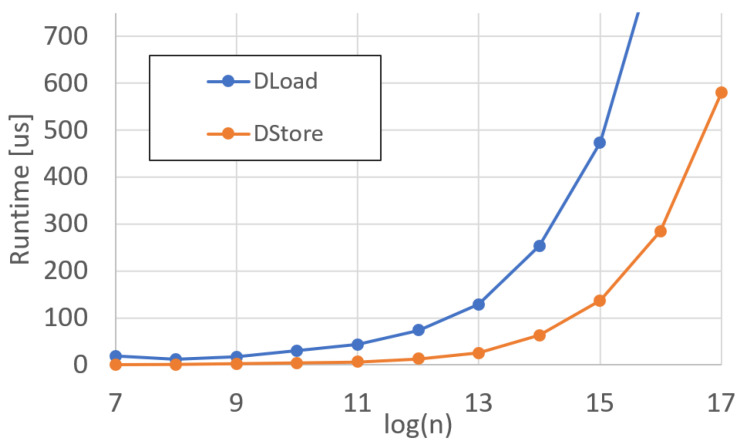
DARTPHROG DMA operations runtime.

**Figure 14:**
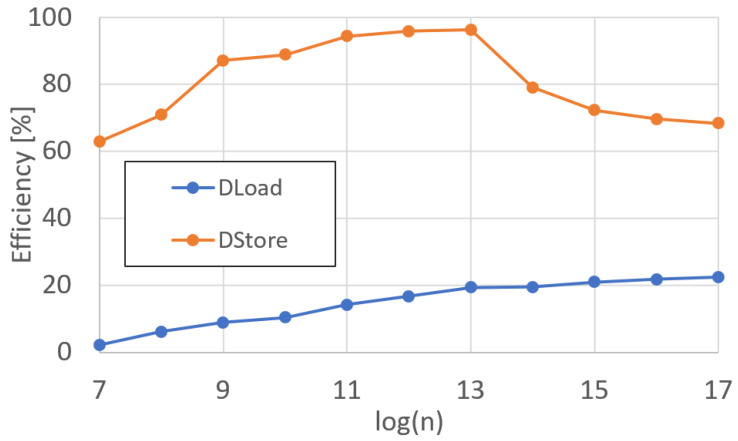
DARTPHROG DMA operations efficiency.

Finally, we measured the runtime of DMA operations for each polynomial of degree *n*. The runtime for loads and stores are displayed in Figure 13, and the efficiency is shown in Figure 14. The results for each functional unit, including DMA operations, are discussed in further detail in Section 5.

## 5. Discussion

Unlike other FHE accelerators that replace open source FHE libraries like OpenFHE and SEAL, DARTPHROG was developed with the means to compliment software libraries by accelerating their primitive operations such as multiplication and subtraction. In fact, DP relies on these software libraries for processes such as the NTT, relinearization, and bootstrapping.

This is in part due to the area and resource limitations from the Alveo U50 card, with less than half of the resources available compared to the more popular U250/U280, and also partly due to power constraints. The U50 has a power limitation of 75 W, as it is only powered via the PCIe slot. Alternatively, the U280 has an upper limit of 225 W. This power level is available through an on-card power connector that allows the draw power directly from a Power Supply Unity (PSU).

We tested the power maximum of the U50 by attempting to double the width of the data bus. Preliminary place and route results demonstrated that this endeavor was infeasible, as predictions showed a power draw of approximately 82 W, which is well beyond the capabilities of what can be supplied through the PCIe card edge. Furthermore, resource limitations also restricted the effort, as pipeline congestion hindered timing closure. For these reasons, we compare our results against software implementations rather than larger FPGA alternatives to DP.

Due to the limitations in our study, power measurements were conducted via estimations from the Vivado power management tool. These estimations are derived from predicted switching levels within the logic cores, such as the ALU and control unit. Furthermore, the power management tool integrates known power requirements from the hard IP cores, such as the integrated HBM controller and PCIe transceiver. Alternatively, power estimations for individual operations, such as additions and multiplications, as well as for entire homomorphic programs, could be made with a PCIe interposer. The interposer could be used to directly inject and monitor power into the Alveo card with controls configured to measure power consumption based on individual DISA instructions. This would allow for more precise power measurements.

### 5.1. Software Comparison

There are multiple open source libraries available for FHE, including Microsoft SEAL, Homomorphic Encryption Library (HELib) [45], and OpenFHE [46], which is the successor to PALISADE.

SEAL is one of the popular libraries, and is still receiving updates from Microsoft at the time of writing of this article. SEAL is implemented primarily in C++ and supports Homomorphic arithmetic, but does not support comparisons, sorting, or regular expressions. The documentation describes SEAL as having a steep learning curve, but with a relatively simple API. Supported schemes from SEAL include BFV, BGV, and CKKS. For the purpose of comparing the speedup of DP, we run a full comparison of the supported functions against the SEAL library.

Alternatively, HELIb, another C++ open source library, supports both BGV and CKKS. Dissimilar from SEAL, HELib supports a unique assembly language targeted towards HE, which allows for simplified multi-threading, noise management, and low-level arithmetic.

OpenFHE takes a broader approach to FHE, implementing the BFV, BGV, and CKKS schemes in C++, like HELib and SEAL. Meanwhile, OpenFHE also includes Ducas–Micciancio, Chillotti–Gama–Georgieva–Izabachene and Lee–Micciancio–Kim–Choi–Deryabin–Eom–Yoo schemes for Boolean circuits. This wider level of support may appeal to some developers, while others may see the extra functionality as unnecessary overhead.

Recent works compare the efficiency of these different libraries against each other. A work by Faneela et al. explores the throughput and memory utilization of SEAL against OpenFHE for various arithmetic functions [47]. This work showed that OpenFHE consistently outperformed Microsoft SEAL, but only by margins of 5–10%.

Another work by Zhu et al. [48] did not compare the benchmark results directly, as in the case of the work by Faneela. Instead, Zhu implemented a neural network in SEAL, OpenFHE, and HELib and measured the results from each. Contrary to the findings from Faneela, Zhu’s work showed the SEAL outperformed OpenFHE by more than 50%. Their work also showed that SEAL had 17% less latency compared to HELib.

Based on these two works, it is demonstrated that SEAL will, at best, outperform the other major open source libraries. In the worst case, SEAL will perform slightly worse than the other libraries. Either way, all libraries perform at similar magnitudes. As we will later show in this section, our work outperforms Microsoft SEAL by more than 1800x. We can extrapolate this to show that our work will have similar performance metrics to OpenFHE and HELib.

For a fair comparison of the opensource Microsoft SEAL library against DP, we modified the standard benchmarking procedures in SEAL software version 4.1.2. The benchmarks were rewritten to operate on polynomials with 256-bit coefficients, with each coefficient being cast in the RNS domain with the same eight 32-bit limbs as DP. Further, benchmarking for each function ensured that the respective ciphertexts and plaintexts were already in NTT form before being operated on.

**Figure 15:**
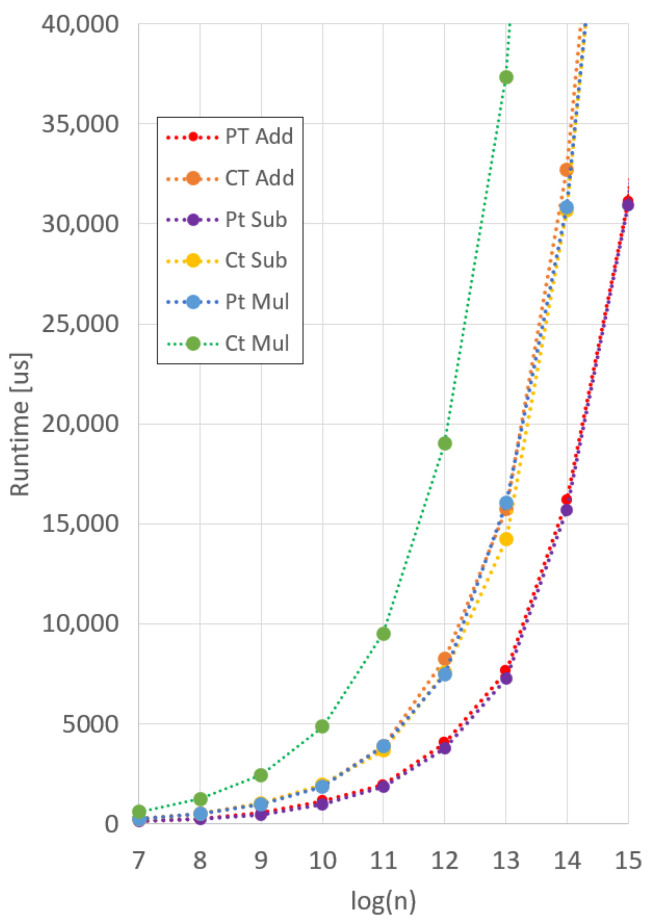
Microsoft SEAL operation runtime.

**Figure 16:**
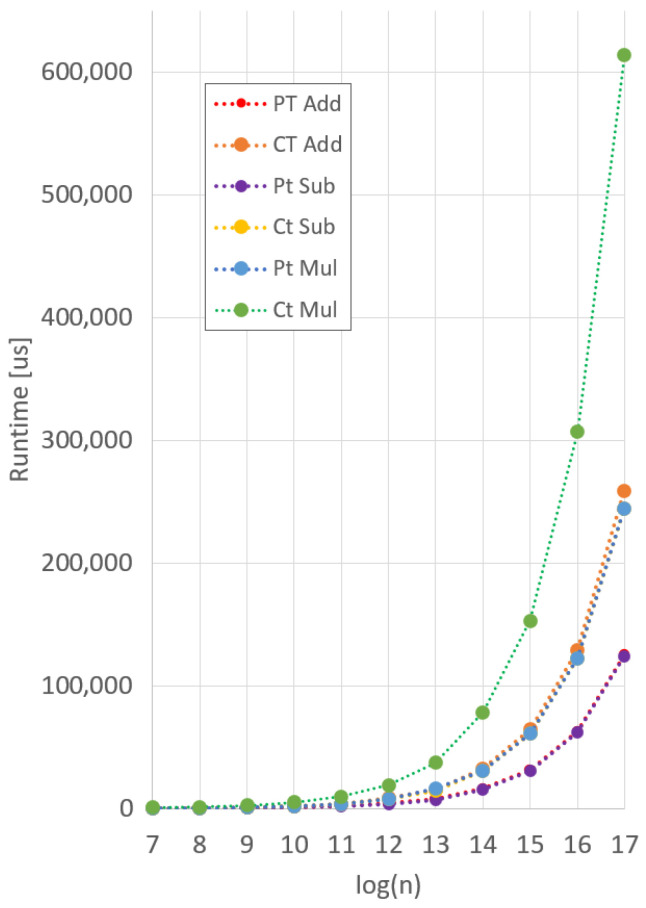
Extrapolated Microsoft SEAL operations runtime.

We ran each benchmark on a 13th generation Intel i7-1360P processor with 32 GB of RAM. To ensure consistent results, each function was averaged over 100 separate runs. As Microsoft SEAL only natively supports ciphertexts up to $2^{15}$ coefficients, we extrapolated data for coefficient sizes of $2^{16}$ and $2^{17}$. This is appropriate, as the trendline for each operation follows a linear increase with the increase in coefficients. Results for the Microsoft SEAL benchmarks are shown in Figure 15, and a zoomed out, extrapolated version is shown in Figure 16.

### 5.2. Results Comparison

**Figure 17:**
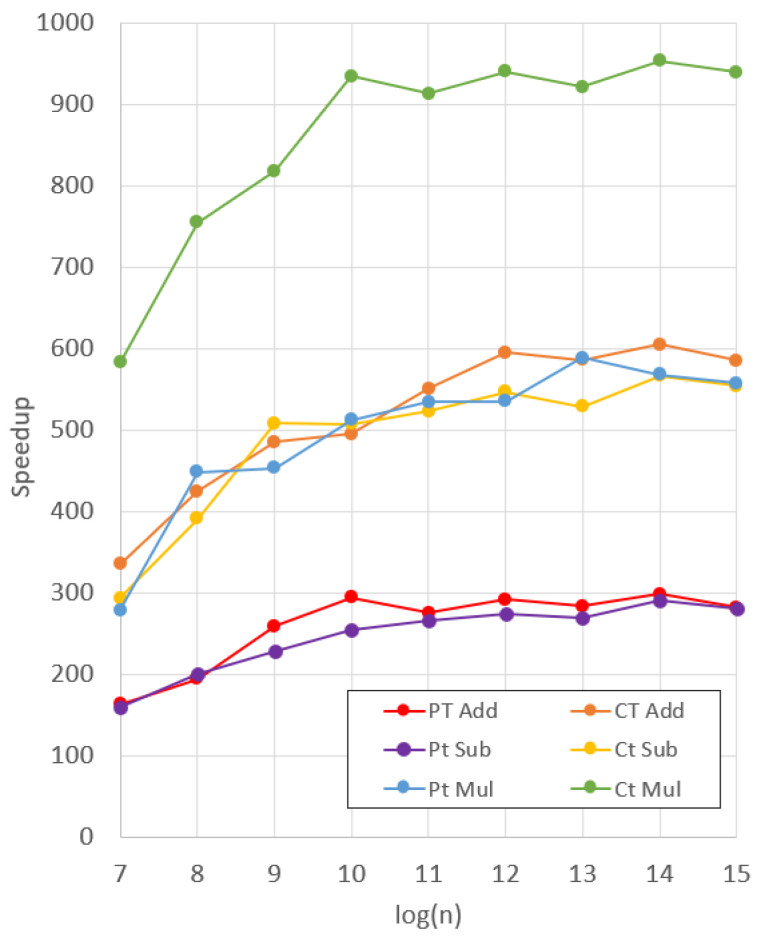
Extrapolated Microsoft SEAL operations runtime.

We display a visualization of the speedup between SEAL and DARTPHROG in Figure 17, and the explicit comparison is listed in Table 5. Between DARTPHROG and SEAL, both versions show a linear relationship between the degree *n* and the runtime for each primitive function, as to be expected with the NTT. However, the rate of change as the degree increases is higher for Microsoft SEAL, leading to higher degree operations to be relatively more expensive to their DP counterpart.

Furthermore, we see the lowest speedup for the plaintext addition and subtraction operations. This is due to the data storage mechanism within DP, shown in Figure 6 for plaintext. In order to make the multiplication of ciphertext/plaintext more efficient, two copies of the plaintext are stored side-by-side, in what would be (c1,c0) if the plaintext were instead a ciphertext. For basic addition and subtraction operations, the upper half of the plaintext is masked, reducing the throughput by half, as the adder is calculating (c1+0,c0+pt). However, this leads to further increases in the PMult operation, showing similar levels of speedup compared to the Ct Add and Ct Sub operations.

The best gains, by a significant margin, are shown in the Ct Mult operation, with a speedup of 580x to 929x that of SEAL. This is explained by the architecture of the Ct Mult Primitive in DP. While Ct Mult requires two multiplications and an addition in the case of d1, DP can calculate the result in a sequential manner without having to store the two products before adding them together. This is not true for SEAL, as a conventional CPU will require load and store operations between each calculation.

Moreover, the speedup can be doubled with a creative tweak to the DP microcode. Note that DP is a superscalar architecture, able to utilize instruction-level parallelism. Further, the requirement that input data be in evaluation form, and that c1 be stored side-by-side c0 in the form of Figure 4 means that a ciphertext or plaintext can be split between two Polynomial registers. By writing the value of *n*/2 to the polynomial size register, one can store a split ciphertext or plaintext into two registers, and another ciphertext or plaintext into two other registers. The two results, whether they be products, sums, or differences, can then be calculated in parallel, and written to two different destination output registers. The destination registers can then be read sequentially, giving the final result. This leads to a doubling in throughput, which is almost an 1860x speedup over Microsoft SEAL. In this way, the results of Table 5 can be doubled. If the programmer wants to turn off this parallelism and revert operations back to the normal size of *n*, they only need to write the value of *n* to the polynomial size register before continuing with their program.

Finally, the results of the DMA operations must be discussed. The DMA load operation, being the H2C operation, is the most inefficient one of all operations within DP. This has a simple explanation: the host cannot transfer data until DP is ready to accept that data. Before it can be ready to accept data, DP must run the loaded program until it reaches a DMA load instruction. Because of this, the host must poll the DP `ready’ register. Once that register is asserted, the DMA engine on the host server can begin transmitting data, whether that data be ciphertext or plaintext. The low efficiency seen in the DMA load operation is due to this process. The wait time for the server to begin loading the data is most significant with small polynomials, as once the server begins transmitting data, it is a continuous stream, increasing the efficiency for larger degree polynomials.

This is not the case for store operations. Since the host server knows exactly how many store operations are going to occur per homomorphic program on DP, the descriptors for moving data off the card can be preloaded in the C2H queue for the card to consume whenever it is ready, effectively removing the wait time seen in the DMA load operation.

The cost for DMA loads and stores must be accounted for when loading HE programs to DARTPHROG. The overhead of DP may be too much for small FHE programs, leading to DP being ideal for longer FHE programs requiring minimal relinearization or bootstrapping operations. To help support this, one should lean towards avoiding ciphertext/ciphertext multiplications when possible, as these operations require relinearization and can quickly increase the noise level.

## 6. Conclusions

Within this work, we presented our custom architecture for accelerating Fully Homomorphic Encryption: the Dynamic Accelerator for Parallel Homomorphic Programs, DARTPHROG. DARTPHROG allows for up to two parallel operations between plaintexts and ciphertexts, including multiplications, additions, and subtractions. Our results showed anywhere between a 150x and 1860x speedup in operations when compared against Microsoft SEAL and the superscalar feature is leveraged to double throughput. Our method implemented the newly published HOM-R method, a Modular Reduction algorithm designed specifically for digital hardware. The resource efficiency of HOM-R allows for parallel, distributed Modular Reduction primitives.

Throughout the development of DARTPHROG, we found the boundaries of the Alveo U50 card. Different areas of exploration led to limitations from power, area, resources, and routing capabilities. We would like to see DARTPHROG extended to larger cards, such as the U250 or U280. We believe that while DP would benefit from a wider data bus or more primitive operations, it would be more benefit to instead committing the extra resources into supporting the NTT. On-chip NTT would mean that bootstrapping and relinearization could take place on the card, and that data would not have to be stored into host memory until the FHE program was completed.

Further, we believe that, the HOM-R system could be extended to support modular switching, a common method in FHE cryptosystems. As it stands currently, the modular bases for HOM-R are written via compile-time parameterization. We would like to see future versions of HOM-R that instead write the lookup tables via registers accessible via PCIe, leading to runtime-configurable modular bases. If this were to be implemented, a programmer utilizing DP could swap the modular bases on the fly, even if they were in the middle of evaluating an FHE program on DP.

## Figures and Tables

**Table 1:** The DARTPHROG ISA, summarized by five general instructions.

	Function	15	14	13	12	11	10	9	8	7	6	5	4	3	2	1	0
LD	Load	1	1	0	0	1						PL			Rd		
ST	Store	1	1	0	0	0						PL			Rd		
ADD	Add	1	0	0	0		Rn				Rs				Rd		
SUB	Subtract	1	0	0	1		Rn				Rs				Rd		
MUL	Multiply	1	0	1	0		Rn				Rs				Rd		

**Table 2:** DARTPHROG resource utilization in terms of total resources utilized.

		LUTs	FFs	BRAM	DSPs
ALU		122,011	259,262	0	384
	Inverter	2309	2070	0	0
	Adder	2060	3723	0	0
	PMult	3157	8288	0	64
	CMult	15,534	20,664	0	128
	Bus Arb.	75,891	189,772	0	0
Reg. Bank		158,083	439,571	6	0
Control Unit		748	2041	0.5	0
XDMA		49,469	51,795	76	0
Misc.		15,916	12,952	59	0
**Total **		**342,227**	**765,621**	**141.5**	**384**

**Table 3:** DARTPHROG resource utilization relative to the total available resources on the Alveo U50 Virtex FPGA.

		LUTs	FFs	BRAM	DSPs
ALU		14%	15%	0%	6.5%
	Inverter	0.27%	0.12%	0%	0%
	Adder	0.24%	0.21%	0%	0%
	PMult	0.36%	0.48%	0%	1.1%
	CMult	1.8%	1.2%	0%	2.2%
	Bus Arb.	8.7%	11%	0%	0%
Reg. Bank		18%	25%	0.0045%	0%
Control Unit		0.0009%	0.12%	0.00037%	0%
XDMA		5.7%	2.3%	5.7%	0%
Misc.		1.8%	0.74%	4.4%	0%
**Total**		**39%**	**44%**	**11%**	**6.5%**

**Table 4:** HOM-R utilization in DARTPHROG.

	LUTs	FFs
Total Adder HOM-R	1503	2649
Average	94	166
Total PMult HOM-R	5935	7358
Average	371	459
Total CMult HOM-R	8951	11,064
Average	374	461

**Table 5:** Speed up of DARTPHROG over Microsoft SEAL.

log2(n)	PT Add	CT Add	Pt Sub	Ct Sub	Pt Mul	Ct Mul
7	164	336	160	294	279	583
8	195	425	201	392	449	755
9	260	486	229	509	454	819
10	295	496	256	508	513	936
11	276	551	267	523	536	914
12	292	595	274	548	536	941
13	284	587	270	529	589	922
14	299	606	291	568	569	954
15	283	586	281	555	558	940
16	287	593	284	560	574	947
17	286	591	284	559	568	929

## Data Availability

Derived data supporting the findings of this study are available from the corresponding author on request.
